# Editorial: What does human pathology bring to the understanding of the fundamental mechanisms of development?

**DOI:** 10.3389/fnana.2022.1003607

**Published:** 2022-08-23

**Authors:** Alfonso Represa, Salvador Martinez, Antoinette Gelot

**Affiliations:** ^1^Aix-Marseille University, INSERM, INMED, Marseille, France; ^2^Instituto de Neurociencias, UMH-CSIC, Alicante, Spain; ^3^Centro de Investigación Biomédica En Red en Salud Mental-CIBERSAM-ISCIII, Valencia, Spain; ^4^APHP, Hôpital Trousseau, Université Pierre et Marie Curie, Paris, France

**Keywords:** neuropathologic findings, brain development, neurodevelopmental disorders, brain malformations, mTOR, interneuron

Brain development involves complex processes that are particularly vulnerable to environmental and genetic factors and that can lead to a wide spectrum of malformations and disturbances, often associated with serious clinical morbidities. It is thus estimated that ~3 in 1,000 pregnancies are associated with embryonic brain malformations. Despite fetal ultrasound and MRI imaging techniques, which have known an extraordinary progress allowing the diagnosis of quite early brain malformations, neuropathological analysis remain a powerful tool for the diagnosis of fetal brain abnormalities providing important cellular cues for understanding their pathogenic mechanisms and etiologies. In addition to this, histopathological analysis of the altered brain combined with genetic and experimental investigations, what is illustrated by the growing number of publications combining fundamental, genetic and histopathological approaches, also improve our understanding of normal human brain development.

The objective of this topic was to reflect through a few examples, the way in which experimental research and neuropathological research combine today in a complementary way, helping us to better understand the normal and pathological development of the brain.

The topic includes five review papers and three original contributions.

Feliciano presents an excellent updated revision of the pathogenesis of Tuberous Sclerosis Complex (TSC), a rare, multi-system genetic disease characterized by the development of non-cancerous (benign) tumors to grow in the brain and on other vital organs resulting in a combination of symptoms including seizures, intellectual disability, developmental delay, skin abnormalities, and kidney disease. This disease is caused by mutations on TSC1 or TSC2 genes, which act as growth suppressors by inhibiting the activation of mTOR (mechanistic target of rapamycin), which regulates multiple cellular functions, such as cell metabolism, growth, proliferation, and survival. Feliciano's review is complemented by a fairly comprehensive evaluation of TSC animal models which illustrates well how human pathology can provide an understanding of the fundamental mechanisms of development.

Gelot and Represa, present in their manuscript original data obtained from the analysis of 16 fetal brains cases of TSC covering the developmental period between 19 gestation week and the 8th post-natal month of life. Their data support the notion that in addition to the well-documented implication of cortical projection neurons, cortical interneurons also contribute to the development of tuberal lesions. First, dysplastic cells invade the cortical plate from superficial (the marginal zone) and deep (the periventricular zone) zones and second, parvalbumin and calbindin immunoreactive cells display dysmorphic and cytomegalic features in cortical tubers and subcortical nodules. Thus, TSC lesions should be considered as mosaics composed of different cell lineages that develop abnormal features as a result of loss of function mutation of TSC genes.

The mTOR signaling pathway is implicated in many other brain alterations, including Hemimegalencephaly and Focal Cortical Dysplasia type II, conditions that obviously share many similarities in their pathogenesis with TSC. Nguyen and Bordey reviewed in their manuscript the link between mTOR-related brain alterations and epileptogenesis, primarily analyzing animal models induced by *in utero* electroporation. The authors discuss at length the molecular origin of brain alterations and functional changes leading to hyperexcitability. They conclude their review with a very interesting discussion on the therapeutic perspectives of epilepsy associated with these pathologies.

Cachia et al. review in their manuscript the characterization and development of the sulcal/sulco-gyral pattern of the cortex, that has been evaluated by pathologist before and that is now more systematically investigated and quantified by MRI. Their article discusses at length literature indicating that sulcal features would determine future cognitive abilities, like cognitive control or reading abilities. In addition, their article reviews studies measuring sulcation patterns in patients with schizophrenia and affective disorders, studies suggesting that early developmental variations may be one of the factors contributing to clinical manifestations or risk factor for developing this type of symptomatology. Thus, it was proposed that sulcal pattern, which are determined before birth and stable throughout life, might constitute transdiagnostic trait marker of cerebral dysfunction.

The work of Garcia-Lopez et al. demonstrates the involvement of interneurons in the phenotype of type 1 lissencephalies linked to LIS1 mutations and hypothesizes that this would the missing link between the genetic variations in the lissencephalic critical region (17p13.3) around LIS1 gene observed in schizophrenia patients and the schizophrenia phenotype. Beyond that, this work highlights the hitherto underestimated importance of the role of interneurons in cerebral malformations.

The development of the craniofacial complex is tightly connected with the development of the brain mainly though the role of the cranial neural crest cells and complex molecular crosstalk between the two. Accordingly, facial malformations are often associated with defects in the underlying brain. Sándor-Bajusz et al. analyze in their manuscript the link between non-syndromic oral cleft and brain structural variances. The meta-analysis they performed indicates that oral cleft subjects display smaller cerebral gray matter, cerebellum, temporal lobes, and occipital lobes when compared to controls. Authors carefully discuss the risk of bias but highlight the interest of the observations that can contribute to neuropsychiatric disorders frequently reported in patients with non-syndromic oral clefts.

Stonebridge et al. describe the discovery of an enlarged AEAF during a conventional autopsy. Their work underlines several points: (i) the interest of post-mortem examination, which one might think unnecessary in the age of imaging and genetics; (ii) the demonstration that neuropathological examination is complementary of other diagnostic techniques; and (iii) the contribution of human pathology to the exploration of both pathophysiological and developmental mechanisms.

Diaz and Puelles provide a comprehensive review of the mapping of hypothalamic morphogens. This work is also an opportunity to discuss the historical evolution of the concepts of brain developmental processes as well as the contribution of human pathology to the understanding of developmental mechanisms.

We hope that this topic shows that current knowledge on brain development, resulting from basic research, helps a better understanding of the histopathological changes observed in developmental disorders, but also illustrates how histopathological analysis continues to irrigate our knowledge of developmental processes through strong interactions with other disciplines, helping us to reformulate fundamental questions.

## Author contributions

All authors listed have made a substantial, direct, and intellectual contribution to the work and approved it for publication.

## Conflict of interest

The authors declare that the research was conducted in the absence of any commercial or financial relationships that could be construed as a potential conflict of interest.

## Publisher's note

All claims expressed in this article are solely those of the authors and do not necessarily represent those of their affiliated organizations, or those of the publisher, the editors and the reviewers. Any product that may be evaluated in this article, or claim that may be made by its manufacturer, is not guaranteed or endorsed by the publisher.

